# Hydroxyurea Use Among Children With Sickle Cell Disease at King Abdulaziz University Hospital in Jeddah City

**DOI:** 10.7759/cureus.13453

**Published:** 2021-02-20

**Authors:** Fatma Alzahrani, Ghaidaa F Albaz, Fatima AlSinan, Jumana Alzuhayri, Zahra M Barnawi, Nouf Melebari, Tethkar M Al Twairgi

**Affiliations:** 1 Pediatrics, King Abdulaziz University Hospital, Jeddah, SAU; 2 Medicine, King Abdulaziz University Hospital, Jeddah, SAU; 3 College of Medicine, Imam Abdulrahman bin Faisal University, Dammam, SAU; 4 Faculty of Science: Biochemistry, King Abdul-Aziz University, Jeddah, SAU; 5 Health Education, Umm Al Qura University, Makkah, SAU; 6 General Medicine, Taif University, Taif, SAU

**Keywords:** hydroxyurea, sickle cell disease, hemoglobinopathies

## Abstract

Background

Sickle cell disease (SCD) is an autosomal recessive hemoglobinopathy that is very common and causes a great burden in Saudi Arabia and worldwide. This study aims to determine the frequency and benefits of using hydroxyurea in treating children with SCD in King Abdulaziz University Hospital (KAUH) in Saudi Arabia.

Method

This retrospective observational study included all SCD patients, males and females, who were following up in KAUH and were less than 18 years old. Patients on chronic transfusion therapy or who had previous stem cell transplantation were excluded from the study. The study was approved by the unit of biomedical ethics in KAUH, Jeddah.

Result

The study included 102 SCD patients; the median age of the sample was 7.5±4.2, with the majority being female (58 patients; 56.9%). Hydroxyurea users among participants in the study were 62 patients (60.8%). Thirty-seven (37) of the patients using hydroxyurea had an increase in the mean corpuscular volume (MCV). There was a decrease in the level of white blood cells (WBCs) and neutrophil count from 15.81±10.24 and 48.31±23.23% to 12.48±5.48 and 40.81±15.78%, respectively. Platelet count showed an increase from 345.4±2.1096 to 359.162±199.87 after starting hydroxyurea. The incidence of vaso-occlusive crisis (VOC) increased after hydroxyurea initiation from 4.555±4.08 to 6.288±9.80. Moreover, the frequency of blood transfusion in patients using hydroxyurea was statistically significant as p-value = 0.048.

Conclusion

In conclusion, our results showed significant clinical and laboratory benefits of hydroxyurea in children with SCD. Hydroxyurea has been shown to reduce the frequency of VOC and blood transfusion in patients with SCD.

## Introduction

Sickle cell disease (SCD) is an autosomal recessive hemoglobinopathy that is very common and causes a great burden in the Kingdom of Saudi Arabia (KSA) and worldwide [1-2]. SCD results from a single mutation in the β-globin, which changes the glutamic amino acid to valine forming abnormal sickle hemoglobin (HbS) [3]. Under deoxygenated conditions, HbS will cause the red blood cells (RBCs) to take the sickle form. Aggregation of these abnormal RBCs block the blood vessels, decreasing the blood flow distally resulting in an ischemic tissue injury. Recurrent episodes of sickling will eventually lead to long-term end-organ damage such as vision problems and delayed growth [1-2].

SCD is common worldwide with the highest prevalence being in sub-Saharan Africa, South Asia, Middle East, and Mediterranean countries [4]. The prevalence of SCD in KSA is higher than in other countries, being highest in the Eastern province with a prevalence of 145 per 100,000 population in 2017 [5].

The main modalities for treating SCD include blood transfusion, stem cell transplantation, and medical treatment with hydroxyurea [1]. Hydroxyurea is a chemotherapeutic agent that among other properties increases the level of fetal hemoglobin (HbF), which decreases the sickling of RBCs, therefore, decreasing the incidence of various SCD-related complications [1-3,6-7]. Hydroxyurea has been recently approved by the Food and Drug Administration (FDA) as a treatment for children with SCD [2].

Several clinical trials have demonstrated the significant clinical benefits of hydroxyurea in reducing the frequency of vaso-occlusive crisis (VOC), acute chest syndrome (ACS), blood transfusion, hospitalization, death, and other complications in children with SCD [2,8-11]. Hydroxyurea has also shown significant improvement in different hematological parameters such as hemoglobin, hemoglobin F, mean corpuscular volume (MCV) [8].

It has been documented that hydroxyurea can improve the clinical outcome and hemoglobin level in the real-life setting rather than being effective only in the setting of clinical trials [12]. The studies also show a decrease in the rate of hospitalization, long-term complications, as well as protection against end-organ damage in children taking hydroxyurea [12].

No previous studies have been done addressing the frequency and benefits of using hydroxyurea in Saudi Arabia, which are needed for the healthcare providers to make recommendations to the patients and their families. This study aims to determine the frequency and benefits of hydroxyurea treatment for children with SCD at King Abdul-Aziz University Hospital (KAUH) in Saudi Arabia.

## Materials and methods

This is a retrospective observational study of the frequency and benefit of using hydroxyurea in children who were diagnosed with SCD from 2014 to 2019 in King Abdul-Aziz University Hospital (KAUH) in Jeddah, KSA. The study was conducted between June and July 2019, and it was approved by the unit of biomedical ethics at KAUH.

The target sample included all patients, males, and females with SCD who less than 18 years old. Patients who were on chronic transfusion therapy (defined as monthly transfusions for at least three months) or had previous stem cell transplantation were excluded from the study. One-hundred two patients were initially included. None of them were on chronic transfusion therapy or had previous stem cell transplantation.

After obtaining hospital approval to access the patient’s files, the data were collected from the patient’s medical record into Microsoft Excel (Microsoft Corporation, Redmond, WA). The collection data sheet included age, gender, weight and height, hydroxyurea use, number of previous hospitalizations and emergency room (ER) visits, acute complications encountered during the period of the treatment, as well as the three months before starting it, including a vaso-occlusive crisis, acute chest syndrome, stroke, infections, number of blood transfusion, previous stem cell transplantation, and laboratory values: complete blood count (CBC), reticulocyte count, and hemoglobin electrophoresis.

The data were then analyzed using the Statistical Package for Social Science (SPSS) version 21 (IBM Corp, Armonk, NY). Mean and standard deviation was used to describe the numerical variable. Numbers and percentages were used to describe categorical variables. Independent t-test and chi-square tests were used to measure the association between different variables. All tests were analyzed with a 95% confidence interval. P-value 0.05 and less was considered to be significant.

## Results

Patients characteristics

The median age of the study population was 7.5± 4.2 (ranging between one and 18 years old) (Table 1), with the majority being female (58 patients; 56.9%) (Table 2). Both sickle genotype HbSC and HbSS were included in the sample with 72 patients (70.6%) having the HbSS genotype (Table 2).

**Table 1:** Demographic data for 102 SCD patients SCD: sickle cell disease; BMI: body mass index

Demographics	Mean ± SD	(Min-Max)
Age (years)	7.50+4.2	(1-18)
BMI	15.24+28.50	(6-275)
Weight (Kg)	17.90+ 10.858	(6-55)
Number of blood transfusions	6.50+25.31	(0-132)

**Table 2:** Hydroxyurea use and patient characteristic SCD: sickle cell disease; ACS: acute chest syndrome

Demographics	Hydroxyurea use frequency	P value	Total
Gender			
Male	26	0.760	44 (43.1%)
Female	36		58 (56.9%)
Nationality			
Saudi			47 (46.1%)
Non-Saudi			55 (53.9%)
Sickle genotype			
HbSS	42	0.10	72 HBSS (70.6%)
HbSC	20		28 HBSC (27.5%)
Non-specified			2 (2%)
Using hydroxyurea or not			
Yes			62 (60.8%)
No			40 (39.2%)
UTI			
Yes	18	0.100	26
No	44		74
Stroke			
Yes	13	0.273	18
No	49		84
ACS			
Yes	18	0.103	24
No	44		78

Hydroxyurea use

The hydroxyurea users among the study participants were 62 patients (60.7%); 36 hydroxyurea users were female while 26 were male; however, the difference between males and females was not statistically significant (p values = 0.76) (Table 2).

Laboratory benefits

Thirty-seven patients using hydroxyurea had an increase in the mean corpuscular volume (MCV); this result was statistically significant (p-value=0.011) (Table 3). There was a decrease in the level of white blood cells (WBCs) and neutrophil count from 15.81±10.24 and 48.31±23.23% to 12.48±5.48 and 40.81±15.78%, respectively. The change in WBCs level was statistically significant as p-value=0.000008 (Table 3). However, the platelet count showed an increase from 345.4±2.1096 to 359.162±199.87 after starting hydroxyurea. This increase in platelet count was not statistically significant (p-value=0.22) (Table 3).

**Table 3:** The effect of hydroxyurea on laboratory and clinical parameters HU: hydroxyurea; MCV: mean corpuscular volume; WBC: white blood cell; VOC: vaso-occlusive crisis

Change in laboratory parameter	Before HU use	After HU use	P-value
MCV	78.29+11.7	95.5+81.32	0.011
WBC	15.81+10.24	12.48+5.48	0.000008
Neutrophils	48.31+23.23%	40.81+15.78%	0.592
Platelets	345.4+2.1096	359.162+199.87	0.22
Pain/VOC	4.555+4.08	6.288+9.80	0.869
Blood transfusion			0.048

Clinical benefits

There was an increase in the incidence of vaso-occlusive crisis (VOC) after hydroxyurea initiation from 4.555+4.08 to 6.288+9.80; the results were not statistically significant (p-value= 0.869) (Table 3).

The frequency of ACS was much lower among patients using hydroxyurea. Forty-four patients did not have any episodes of ACS while they were using hydroxyurea as compared with 18 patients who did have ACS while on treatment. However, this result was not statistically significant (p value=0.103) (Table 2).

The mean number of blood transfusion patients received during the treatment was about 6.5±25.31 (Table 1). The relationship between the number of blood transfusion patients required and the use of hydroxyurea was statistically significant as p-value=0.048 (Table 3).

## Discussion

Hydroxyurea use

Hydroxyurea users were the majority of the patients included, which was 62 patients (60.8%). This is similar to the previous study done in V. Cervello Hospital, as more than half of the patients (72%) were using hydroxyurea [11]. A study done in Italy has also reported an increase in hydroxyurea use recently after a change in their national guidelines [10].

Laboratory values

As in previous studies, there was an increase in MCV from 78.29±11.7 to 95.5±81.32 after hydroxyurea initiation, which is an expected result [2,12-13]. This increase in the MCV was statistically significant (p-value=0.011).

Hydroxyurea is a myelosuppressive agent that normally reduces the bone marrow reactively, resulting in various degrees of cytopenia, thus lowering the production of WBC, neutrophils, platelets, and others. This effect of hydroxyurea can also contribute to additional benefits, as it reduces inflammation, hence the adverse event and complications of SCA [6]. Previous studies have shown significant cytopenia in SCD children treated with hydroxyurea [3,11-12].

Similarly, in this study, the WBC count decreased after starting hydroxyurea from 15.81±10.24 to 12.48±5.48, and neutrophils decreased from 48.31±23.23% to 40.81±15.78% (p-value=0.592). However, platelet count showed an increase from 352.35±216.39 to 363.14±207.995, which was unexpected.

Clinical benefit

The frequency of pain crisis has increased from 4.555±4.08 to 6.288±9.80 in patients taking hydroxyurea. This was an unexpected result, as all the previous studies have shown a significant reduction in the incidence of VOC [2,8-10,12-13]. Hydroxyurea works by increasing the fetal hemoglobin (HbF) level, which reduces the red blood cell (RBC) sickling and hence decreases the frequency of VOC. The reduction in neutrophils count also plays a role by liming tissue damage and pain sensation [7].

The data also showed a lower incidence of ACS following hydroxyurea use. This is consistent with previous studies that report a significant clinical improvement in the frequency of ACS after starting hydroxyurea [9,11-12].

There was a significant association between the use of hydroxyurea and the frequency of blood transfusion as the p-value was 0.048. The study done in Atlanta had similarly shown a decline in the rate of transfusion of about 57% [12]. Likewise, the study done in Sub-Saharan Africa showed a decrease from 43.3 to 14.2 per 100 patient-years [2].

Although blood transfusion has been considered one of the primary and most beneficial modalities for the treatment of SCD, long-term and frequent blood transfusion are not regarded to be safe [1,14]. The complications of regular blood transfusions include the transmission of some infections, such as hepatitis B, hepatitis C, and human immunodeficiency virus (HIV), alloimmunization, and iron overload [1]. Therefore, a reduction in the number of blood transfusions is considered beneficial.

Our study had several limitations, including no fixed specific duration and dose between patients using hydroxyurea. Patients who have used hydroxyurea for less than one month are included in the study, so the degree of benefit would be different compared with patients who took hydroxyurea for years. Second, the patient’s compliance with treatment was not measured. Hence, the results might be affected, and the benefits might not be as in compliant patients.

Our study is a retrospective study; this has limited the amount of monitoring during the duration of treatment. Doing a prospective study in the future would allow us to do regular follow-ups and measure the benefits more closely. Future studies should include a larger sample of patients and be done in multiple centers. More studies should be done on the benefits of hydroxyurea according to the dose taken.

## Conclusions

In conclusion, hydroxyurea is still considered the main treatment for SCD. Our results showed significant clinical and laboratory benefits of hydroxyurea in treating children with SCD. Hydroxyurea was found to increase MCV and decrease the WBC and neutrophil count. It also has been shown to reduce the frequency of vaso-occlusive pain crises, acute chest syndrome, and blood transfusion among SCD patients.
